# Severe Raynaud’s phenomenon from ethosuximide raised concern over possible onset of systemic vasculitis: a case report

**DOI:** 10.1186/s12969-022-00782-8

**Published:** 2022-12-22

**Authors:** Lillemor Berntson, Gunnar Liminga

**Affiliations:** Department of Women’s and Children’s Health, Uppsala University, SE-75185 Uppsala, Sweden

**Keywords:** Ethosuximide, Anti-epileptic drugs, Absence epilepsy, Adverse effect, Drug-induced, Vasculitis, Raynaud’s syndrome, Anti-scl-70

## Abstract

**Background:**

Ethosuximide and other anti-epileptic drugs have been reported to cause idiosyncratic reactions such as lupus-like syndromes, with elevated antinuclear antibody (ANA) levels. Herein, we present a case of a girl who developed a very severe Raynaud’s phenomenon reaction and anti-Scl-70 antibodies related to treatment with ethosuximide, due to juvenile absence epilepsy (JAE).

**Case presentation:**

A 12-year-old girl was diagnosed with JAE and treatment with ethosuximide was initiated. Two and a half months later her fingers, digits II–V bilaterally, began to ache and were discolored, alternatingly white, blue, or normal-colored. Two weeks later, her fingers were bluish-black, aching severely, almost continuously. The family sought medical advice. Ethosuximide was halted and due to the severe symptoms, treatment with both prednisolone and intravenous iloprost was commenced. Laboratory tests revealed high ANA levels with anti-Scl-70 pattern and confirmed anti-Scl-70 antibodies. After a few weeks, she started to improve and the symptoms slowly decreased over five months. Anti-Scl-70 was still detectable four months after onset of symptoms, though she was much improved. After eleven months, repeated ANA analyses were completely negative.

**Conclusion:**

Although extremely rare, it is important to recognize that severe Raynaud’s phenomenon, threatening peripheral digital circulation, may occur as an idiosyncratic reaction to ethosuximide, raising concern over possible onset of vasculitis.

**Supplementary Information:**

The online version contains supplementary material available at 10.1186/s12969-022-00782-8.

## Background

Raynaud’s phenomenon is a multifactorial vasospastic disorder characterized by a transient, recurrent, and reversible constriction of peripheral blood vessels. Different forms of Raynaud’s phenomenon (RP) may sometimes be hard to differentiate. Primary RP (i.e., Raynaud’s disease) should be distinguished from secondary Raynaud’s phenomenon (i.e., Raynaud’s syndrome), as long-term morbidity differs greatly between the two conditions [1]. Secondary RP is associated with an underlying disease.

In pediatric rheumatology, RP may occur in various inflammatory systemic diseases, but a more severe presentation is most commonly part of a mixed connective tissue disease or juvenile systemic scleroderma.

One of the drugs occasionally suspected of causing adverse events mimicking a vasculitis-like disease is ethosuximide. Ethosuximide is still the first-line treatment for absence epilepsy that develops during childhood [2]. Together with carbamazepine, phenytoin, primidone, and phenobarbital, it is one of the older anti-epileptic drugs still in use.

Adverse events in ethosuximide treatment have mostly been associated with nausea, vomiting, and behavioral/psychiatric changes [2], but idiosyncratic side effects, such as lupus-like reactions and vasculitis, have also been reported [3–6], although a severe Raynaud’s phenomenon leading to a series of iloprost infusions and concomitant occurrence of anti-Scl-70 antibodies has not been described previously.

## Case presentation

**Fig. 1:**
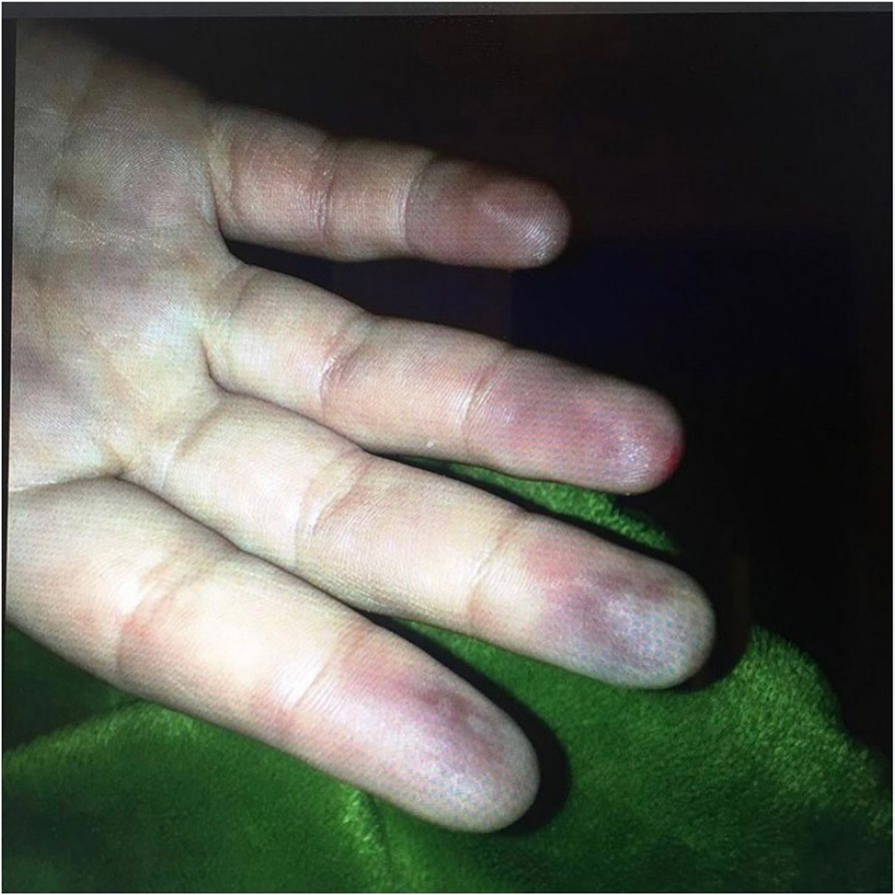
Picture of the fingers and palms of the patient when her circulation was severely affected

We present the case of a girl born to unrelated parents. The father suffered from febrile seizures during childhood and the mother had migraines and tension headaches. Beyond that, there was no medical history of any illness. The girl had a previous medical history of infectious asthma, viral croup, and treatment with tympanostomy tubes because of recurrent otitis media infections. She had no previous history of reactive circulation in fingers or toes. At the age of four years, she had been observed over night because of a suspect vasovagal reaction. At the age of twelve years, friends noticed that she had short periods of absence and she was referred for an electroencephalography (EEG). The EEG showed typical 3 Hz spike-slow wave configurations during photo stimulation and sleep and she was diagnosed with juvenile absence epilepsy (JAE) by a consultant at the pediatric neurology clinic. Treatment with ethosuximide, oral solution 50 mg/ml, was initiated. The starting dose was 7.5 mg/kg/day and the dose increase was halted at 15 mg/kg/day due to nausea, heartburn, and fatigue. Serum concentration was 378 µM (280–700 µM) and the patient was then seizure-free, attending school, and playing soccer. Three months after ethosuximide treatment was initiated and two months after the last dose adjustment, her fingers began to ache (primarily her middle fingers, digit III, bilaterally) and color changes were noticed in her fingers, which alternated between being white, blue, and normal-colored. Two weeks later, her fingertips were cold and had changed in color, her left digits II and III and right digits II–IV were blueish-black distal to the distal interphalangeal joints, Fig. 1. There was no extreme cold exposure or stressful episode prior to those symptoms and no symptoms of any infection. Her mother contacted the epilepsy nurse and the girl was admitted to hospital. Peripheral circulation was affected also in her toes, but to a much lesser extent and nailfold qualitative capillary microscopy was normal. There were no signs of edematous swelling of fingers or presence of inelastic skin, hyper- or hypopigmentation of the skin, joint contractures, or synovitis and no complaints of decreased esophageal motility or breathing problems. At admission, ANA was strongly positive with a pattern specific for anti-Scl-70 (AC-29; see www.anapatterns.org). Occurrence of specific anti-Scl-70/topoisomerase 1 antibodies was independently confirmed with three techniques: double immune diffusion, enzyme-linked immunosorbent assay, and line immunoassay. Anti-Scl-70 and the corresponding ANA pattern were still detected after almost four months. IgG -antibodies against nDNA, Sm, RNP, SSA/Ro52, SSA/Ro60, SSB, and Jo-1 were negative. Rheumatoid factor (IgM), proteinase 3 antineutrophil cytoplasmic antibodies, and myeloperoxidase antibodies were negative as were the antiphospholipid antibodies lupus anticoagulant, anti-cardiolipin and anti-beta-2 glycoprotein-1. The peripheral blood count was normal, but activated partial tromboplastine time (APTT) was increased to 50 s (ref 20–42 s) and increases in complement C3d and the C3d/C3 ratio were found, indicating possible complement activation. See Table 1 for detailed laboratory test results. There was suspicion of an unusual onset of juvenile systemic scleroderma, but also that the symptoms could be caused by ethosuximide. Treatment was stopped and changed to lamotrigine. Due to the severity of the symptoms, the girl received 1 mg/kg of prednisolone for seven days with tapering over four weeks.

**Table 1:** Results from blood analyses performed over four years in an adolescent girl suffering an adverse event after ethosuximide treatment at age 12 years

	At admission	After admission					
Time from admission (months)	0	4	11	14	18	37	49
Peripheral blood count	normal	normal	normal	-	normal	normal	normal
E-SR	3	3	11	-	-	-	-
APTT (ref 30–42 sec)	50	-	45	-	45	-	43
Complement	Increased level of C3d and C3d/C3-fraction	Increased C3d/C3 fraction Possible activation	normal	-	normal	-	-
ANA	Strong homogenous pattern^a^	Strong homogenous pattern^a^	normal	normal	normal	-	-
Anti-Scl-70 antibodies	Positive	Positive	Negative	Negative	Negative	-	-
Albumin (41–50) g/L	38	40	-	-	-	-	-

*E-SR* erytrocyte sedimentation rate, *APTT* activated partial thromboplastin time, *ANA* antinuclear antibodies

^a^Strong pattern refers to a titer of 1:800 or above, detailed explanation in Additional file 1

Seven days after prednisolone treatment was initiated, the pain had started to decrease, but the fingers were still severely discolored. Doppler evaluation of the arterial blood flow in the fingers indicated reduced arterial blood flow in the distal phalanxes of the affected fingers. After discussion with an adult rheumatologist, a vascular surgeon, and a pediatric cardiologist, echocardiography of the heart was performed and found to be normal. Treatment with daily infusions with iloprost for five days was then initiated: 0.9 µg/kg for six hours. The discoloring and symptoms started to disappear, but infusions with iloprost were continued for another five days. Nifedipine 10 mg daily and acetylsalicylic acid 75 mg daily were added after the iloprost infusions. At this point, the severe effect on the digital circulation was considered to be a non-vasculitic vasculopathy, for which reason no more prednisolone was given after the first four weeks.

Approximately 3 weeks after admission, the discoloring and discomfort was very much improved, but the girl developed small ulcers on some of the affected fingertips. Those healed without complication within approximately 2.5 months. Upon evaluation 5 months after the start of treatment, the girl was free of symptoms and sensory function in the hands was normal.

Acetylsalicylic acid was stopped after 4 months and nifedipine was stopped after approximately 6 months. After normalization of ANA, levels remained normal 2.5 years after onset of symptoms, with negative immunofluorescence and anti-Scl-70 undetectable using line immunoassay and addressable laser bead immunoassay on three different occasions. The girl did not have any other signs of systemic sclerosis or systemic lupus erythematosus (SLE).

Today, more than seven years after onset of symptoms, the girl has no signs or symptoms of any rheumatic disease. Her laboratory work-up were normal for another three years and no more blood analyses were performed after that.

## Discussion

We have described a case of severe RP caused by ethosuximide in a child, leading to many months of suffering. She was treated with prednisolone, a ten-day infusion of iloprost, followed by treatment with acetylsalicylic acid and nifedipine for many months. No previous case reports have been presented on RP caused by ethosuximide in children. Therefore, we find it important to raise awareness of this serious side effect.

Already at admission, after two weeks of increasing symptoms, concerns about future damage to peripheral structures in the patient’s fingers were raised. Despite a very severe effect on the digital circulation with limited necrosis on the fingertips, the nailfold capillaries did not show any signs of vasculitis or morphological abnormalities as in systemic scleroderma in our patient. Other signs or symptoms of systemic inflammatory disease were also lacking. Still, introduction of prednisolone after the diagnostic work-up, to decrease the activity of possible vasculitis, was considered inevitable. Pain slowly decreased and prednisolone had a possible, but not proven, effect. The occurrence and verification of anti-Scl-70 antibodies using all three methods described above led to the question if the girl was developing juvenile systemic sclerosis. Double immune diffusion is the most specific for systemic sclerosis of these methods [7]. The results on anti-Scl-70 antibodies arrived 12 days after admission and blood sampling and 12 days after introduction of prednisolone. At that time, the circulatory status of her fingers was improved, but still very poor, and pain had decreased. Doppler evaluation of arterial blood flow indicated that it was reduced in the distal phalanxes of the affected fingers. After consultation with other specialists, iloprost infusions were introduced 15 days after admission. Peripheral circulation slowly improved. We never performed a follow-up arterial Doppler, as the patient did not have any symptoms of reactive circulation at all prior to commencing ethosuximide and the symptoms eventually resolved completely.

In previously published pediatric case reports related to administration of ethosuximide, the children fulfilled the criteria for SLE to varying degrees. Syndromes suggestive of SLE have been presented, including fever, malar rash, arthritis, and lymphadenopathy, as well as cases of pleural effusions and myocarditis or pericarditis [8]. The signs and symptoms most often faded within months after treatment cessation [4, 5, 8] and only in one case included painful, erythematous areas on the fingertips [4]. The impact has been described as having a lupus-like clinical presentation with nephritis, also fading within months [9], though in one case with continuous SLE with nephritis continuing after cessation of ethosuximide [8]. Ethosuximide is one of the drugs known to induce sclerotic lesions in the skin, but this would not lead to RP or positive ANA [10].

Analysis of anti-Scl-70 antibodies with immunodiffusion is useful in distinguishing systemic sclerosis patients from healthy controls [11] and patients with other connective tissue diseases. Anti-Scl-70 antibodies occur in up to 25% of patients with SLE [12]. Antinuclear antibodies may be found in serum from children who are receiving anti-convulsants, but we have not found any reports on the occurrence of anti-Scl-70 antibodies in those children. The concentration of disease-specific antibodies normally stays increased in patients with systemic sclerosis, but may decrease in milder cases [13]. In our patient, they could no longer be detected after eleven months.

Secondary RP has been shown to be associated with microvascular peripheral endothelial dysfunction [14], which is not the case for primary RP. This opens for development of methods to distinguish between primary and secondary RP using quantitative nailfold capillaroscopy [15]. Nailfold capillaroscopy for RP may be helpful in pediatric rheumatology, at least for distinguishing a possible development of systemic scleroderma from other forms of RP, like idiopathic, primary RP, or RP caused by medication. However, capillary density varies with age and studies on normal age-related patterns are lacking [16].

The transient severe RP and Anti-Scl-70 antibodies in our patient raised diagnostic and prognostic questions. Could a transient injury to endothelial cells have led to severe vasoconstriction, or did the anti-Scl-70 antibodies indicate an antibody/antigen reaction causing vasoconstriction? Ultimately, we made the diagnosis of transient vasculopathy in the presence of autoantibodies.

## Conclusion

Ethosuximide may cause severe Raynaud’s phenomenon with a transient increase in anti-Scl-70 antibodies.

## Supplementary Information

**Additional file 1.** Description of ANA methodology in Sweden.

## Data Availability

The datasets generated during the current study are available from the corresponding author on reasonable request.

## Abbreviations

APTT: Activated partial tromboplastin time
JAE: Juvenile absence epilepsy
RP: Raynaud’s phenomenon
SLE: Systemic lupus erythematosus
