# Supplementary material for: Severe Raynaud’s phenomenon from ethosuximide raised concern over possible onset of systemic vasculitis: a case report
Source: Pediatr Rheumatol Online J. 2022 Dec 22;20:120. doi: 10.1186/s12969-022-00782-8 (PMC9783411; doi:10.1186/s12969-022-00782-8)
Supplement: Supplementary file 1 — Additional file 1. Description of ANA methodology in Sweden. [file 12969_2022_782_MOESM1_ESM.docx]

**Additional file 1.** Description of ANA methodology in Sweden.

Like many Swedish accredited clinical immunology laboratories, the Uppsala University Hospital laboratory no longer routinely titrates antinuclear antibodies in its investigations. The cut-off is set at 95% specificity compared with healthy blood donor controls in line with international recommendations (Agmon-Levin et al. Ann Rheum Dis 2014:73:17–23), and stability over time is maintained with a 1:2 titrated internal control sample evaluated at each analysis occasion. Possible antinuclear antibody test results are “negative,” “weak positive” or “strong positive.” “Strong positive” is given in relation to the internal control, corresponding to the staining intensity of the internal control sample dilution that is two titer steps more concentrated than the endpoint dilution of the control, i.e., with a four times higher antibody concentration than the endpoint titer. Currently, the 95% specificity corresponds to a 1:200 serum dilution, and samples are screened at that dilution. A negative antinuclear antibody result thus implies a negative reaction at the 1:200 dilution, whereas weak positive results correspond to titers 1:200–1:400 and strong positive results correspond to titers 1:800 or above.
